# Should the outcome of focal photocoagulation for center-sparing diabetic macular edema require expanding the definition of center involvement?

**DOI:** 10.1038/s41598-019-41612-4

**Published:** 2019-03-26

**Authors:** Selma Alin Somilleda-Ventura, Dulce M. Razo Blanco-Hernández, Surisadai Serafín-Solís, Virgilio Lima-Gómez

**Affiliations:** 1 Research Direction, Hospital Juarez de Mexico, Mexico, Mexico; 2 Research Division, Hospital Juarez de Mexico, Mexico, Mexico; 3 Ophthalmology Service, Hospital Juarez de Mexico, Mexico, Mexico

## Abstract

Photocoagulation may still be a therapeutic choice for center-sparing diabetic macular edema. We compared the visual evolution after photocoagulation, in eyes with focal diabetic macular edema, stratified per the location of thickening with optical coherence tomography. We evaluated people with type 2 diabetes and focal diabetic macular edema, before and three weeks after focal photocoagulation. We divided the sample by edema location: central (group1); paracentral (group 2) and pericentral (group 3) and compared the proportions of eyes with baseline visual impairment, visual improvement, and visual deterioration between groups; central edema was evaluated with logistic regression, as an explaining variable of baseline visual impairment and visual improvement. The study included 160 eyes: 77 in group 1, 20 in group 2, 63 in group 3; baseline visual impairment was more frequent in groups 1 and 2 (52.6%) than in group 3 (28.6%, p = 0.002, OR 2.77) and as common in groups 1 (51.9%) and 2 (55.0%, p = 0.8). The proportions of visual improvement and visual deterioration did not differ between groups (p > 0.05). The outcome after focal photocoagulation was similar in paracentral (considered center-sparing) and central macular edema; the definition of center involvement, which needs intravitreal antiangiogenics, should expand to include paracentral thickening.

## Introduction

Macular edema is the most frequent cause of visual loss in subjects with diabetic retinopathy^1^; antiangiogenic drugs have replaced photocoagulation to treat center-involving edema because they achieve a larger reduction of retinal thickness and improve visual acuity^2^. Focal photocoagulation is currently indicated for cases of edema with focal leakage outside the central 1,000 μm of the macula, when they have a risk of visual loss^3^.

The Early Treatment Diabetic Retinopathy Study found that eyes with clinically significant macular edema had a higher risk of moderate visual loss; the definition includes cases with central thickening, eyes with “exudates at or within 500 μm of the center, with thickening of the adjacent retina” and those with “a zone of thickening larger than 1 disc area, if located within 1 disc diameter of the center of the macula”^4^. Both center-involving (which affects the central 1,000 µm of the fovea) and center-sparing edema (outside the central 1000 µm of the fovea) have a risk of visual loss, which increases when the thickening includes the center; when center sparing edema is caused mainly by individual leaking microaneurysms (focal edema)^5^, it can be treated with focal photocoagulation.

The outcome of focal photocoagulation in center-sparing edema has not been evaluated using imaging tools, and it would be useful to learn whether all center-sparing edema has similar results after focal photocoagulation, especially in settings where antiangiogenics are unavailable and laser treatment could still be a valid treatment.

Optical coherence tomography quantifies and localizes macular thickening. The macular map divides the macula in nine fields: one central with 1,000 μm diameter, four localized between 500 and 1,500 μm from the macular center and four more localized between 1,500 and 3,000 μm from the macular center^6^. Eyes with center-sparing clinically significant macular edema could have a different evolution after focal photocoagulation if their thickening were adjacent or distant from the macular center, which would require a change in their treatment.

We compared the evolution of visual acuity after focal photocoagulation between subjects with and without center involvement, who had clinically significant diabetic macular edema with focal angiographic leakage.

## Material and Methods

Non-experimental, longitudinal, comparative, retrospective study in subjects with clinically significant macular edema, from Mexico City and its metropolitan area; the available population were people with type 2 diabetes and focal macular edema, treated with focal photocoagulation in a general hospital from August 2008 to July 2014. The study lasted from August 1^st^, 2013 to December 31^st^, 2016, it adhered to the tenets of the Declaration of Helsinki and received authorization from the hospital’s Institutional Review Board (Comité de Investigación y Comité de Ética en Investigación del Hospital Juárez de México).

Inclusion criteria were: people with type 2 diabetes, from any gender, aged 40 to 80 years, with any retinopathy level, who had clinically significant macular edema according to the ETDRS criteria, with focal angiographic leakage, who had a record of best corrected visual acuity and a 6-mm optical coherence tomography fast macular map, on the day of photocoagulation and three weeks after it, who had a spongiform thickening pattern in the optical coherence tomography, and who accepted to participate in the study and signed an informed consent. Exclusion criteria were other retinal diseases that reduced visual acuity, cystic, retinal traction or neurosensory detachment patterns in the optical coherence tomography and macular maps with lousy quality or measurement errors; people who withdrew their informed consent were eliminated from the study.

**Figure 1:**
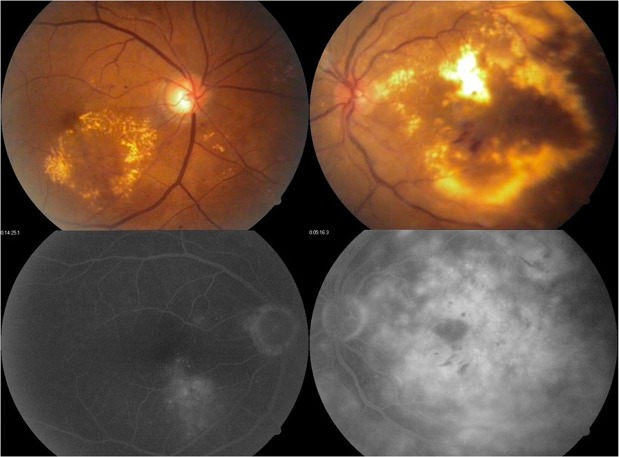
Examples of focal leakage coming mainly from microaneurysms, without mid phase or late fluid accumulation caused by retinal pigment epithelial dysfunction (left). The disfunction of the retinal pigment epithelium shows damage to the external blood-retinal barrier, which causes leakage whose source is not a microaneurysm.

The operative definition of focal edema was any thickening that was caused by focal leakage in fluorescein angiography, which needed to be mainly the result of individual leaking microaneurysms. We considered eyes with any leakage that was not caused mainly by leaking microaneurysms, or included fluid acummulation in mid and late phases resulting from retinal pigment epithelial dysfunction, as having difusse edema and did not include them in the study^5^; (Fig. 1) cases with mixed focal and diffuse edema were not included in the study.

One researcher measured best corrected visual acuity under subjective refraction, in logarithms of the minimal angle of resolution (logMAR). Another researcher, retina specialist, diagnosed clinically significant macular edema using contact lens biomicroscopy under mydriasis, according to the ETDRS criteria: thickening within 500 μm from the center of the macula, exudates at or within 500 μm of the center of the macula, with thickening of the adjacent retina, or a zone of thickening larger than 1 disc area, if located within 1 disc diameter of the center of the macula.

A third researcher obtained a macular map to measure retinal thickness, with the time-domain optical coherence tomography Stratus equipment (Zeiss,Dublin CA, software version 4.0.1). The standardized operative procedure involved: drug mydriasis ≥6 mm, including the anteroposterior visual axis and spherical equivalent, measurement for dark eyes, and optimization of polarization and the z-axis. Centering criteria were a standard ratio deviation of the center point thickness/center point thickness <0.1, and the location of the thinnest zone of the map within the central field^7^.

The investigator who diagnosed clinically significant macular edema treated all the patients with focal photocoagulation, with the following parameters: a 100 μm spot size, a 100 ms duration, and the power required to blanch the leaking microaneurysms at the zones of thickening, located between 500 and 3,000 μm from the center of the macula^8^, using an infrared diode laser (Visulas, Zeiss, Dublin CA). Visual acuity and retinal thickness were measured again, three weeks after treatment; subjects with proliferative diabetic retinopathy received panretinal photocoagulation after obtaining the second macular map at the three-week follow up visit.

**Figure 2:**
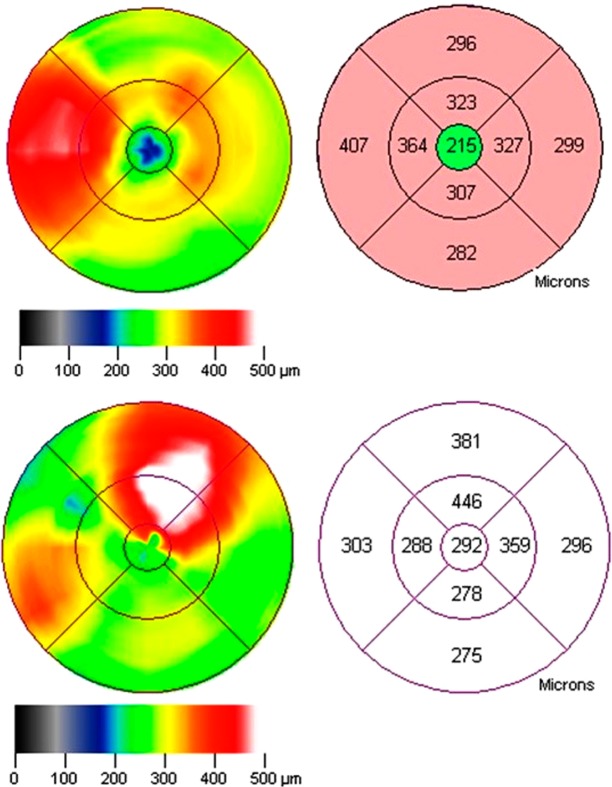
Example of a parafoveal, which increases the thickness of inner and outer ring fields without involving the center (top). The left side of the lower image shows a perifoveal edema, which increases the thickness of an outer ring field; on the right is a large thickening, which involves the inner and the outer ring fields and extends to reach the center, fulfilling the operative definition of center involvement.

We divided the sample according to the location of thickening. Central edema (group 1) included the eyes with thickening at or within 500 μm from the center of the macula, which is the central 1 mm diameter field of the optical coherence tomography map. We classified the eyes with thickening outside the center either as having paracentral (group 2) or pericentral edema (group 3); group 2 included eyes with thickening between 500 and 1,500 μm from the macular center, group 3 comprised all the eyes with thickening found farther than 1,500 μm from the macular center. In the macular map, group 1 had thickening at the central field or center point thickness enlargement; subjects in group 2 had thickening at any of the inner ring fields of the map and eyes in group 3 edema had thickening at any of the outer ring fields (Fig. 2).

We operatively defined thickening as a retinal thickness that exceeded by at least two standard deviations, the mean reported in eyes without diabetic retinopathy from the reference population:

- Central: center field thickness >212.5 µm or center point thickness >187.4 µm
- Paracentral: thickness >301 µm at field 2, >283.3 at field 3, >300.1 µm at field 4 or >302.3 µm at field 5, without thickening of the center point or the center field.
- Pericentral: thickness >277.3 µm at field 6, >259.4 µm at field 7, >262.7 µm at field 8 or >287.8 at field 9, without thickening of the center point, at the center field, or at any inner ring field^9^.

Outcome variables were baseline visual impairment, visual improvement after treatment, and visual deterioration after treatment; their operative definitions were: best corrected visual acuity <0.3 logMAR (20/40 Snellen) before treatment, the gain of at least one line of vision three weeks after focal photocoagulation and the loss of at least one, three weeks after therapy respectively.

We compared visual acuity, center point thickness, center field thickness and macular volume between groups, before and after treatment, using a Kruskal-Wallis test; intragroup variable changes were analyzed using Wilcoxon’s t-test. We also compared between groups the proportions of baseline visual impairment, visual improvement, and visual deterioration using a χ^2^ test and calculated the odds ratio (OR) or the relative risk (RR) when a statistical difference existed. Another evaluation compared group1 against groups 2 and 3 together.

Additionally, a logistic regression analysis was conducted to identify the value of central edema, and central and paracentral edema together, as explaining variables of baseline visual impairment and visual improvement. Gender, arterial hypertension, baseline visual impairment proliferative retinopathy, center point thickening, center field thickening and macular volume enlargement (>7.71 mm^3^)^9^ were covariables in the analysis.

A fourth researcher conducted statistical analysis and considered a p-value < 0.05, an OR or a RR >3 as significant differences; we used the IBM SPSS statistical software version 22, to store and analyze data.

## Results

The study evaluated 160 eyes of 112 patients, aged 41–78 years (mean 56.71 ± 14.01); eighty-eight eyes were from females (55%). Diabetes duration mean was 14.81 ± 6.72 years; in 82 eyes, the patient received treatment with oral hypoglycemic drugs (73.2%). Mean fasting glycemia was 168.3 ± 87.22 mg/dl; seventy-two eyes were from patients with arterial hypertension (45%).

Mean visual acuity before treatment was 20/40 (0.35 ± 0.29 logMAR). Diabetic retinopathy level was mild non-proliferative in 23 eyes (14.4%), moderate non-proliferative in 79 (49.4%), severe non-proliferative in 12 (7.5%) and proliferative in 46 (28.7%).

**Figure 3:**
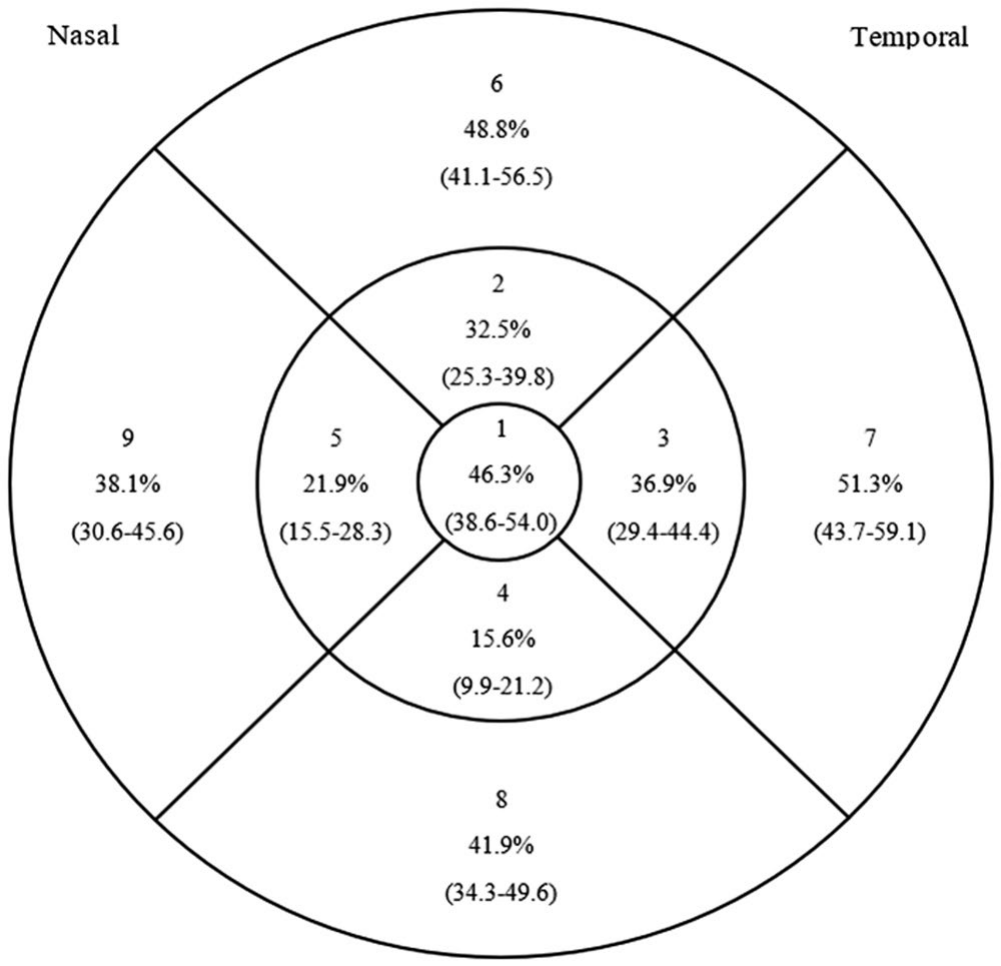
Proportion of eyes with thickening in each field.

Figure 3 shows the proportion of eyes with thickening in each field; there was center point thickening in 61 eyes (38.1%, 95% confidence intervals [C.I.] 30.6–45.6) and macular volume enlargement in 85 (53.1%, 95% C.I. 45.5–60.8).

Macular edema was central in 77 eyes (group 1, 48.1%, 95% C.I. 40.36–55.84) paracentral in 20 (group 2, 12.5%, 95% C.I. 7.38–17.62) and pericentral in 63 eyes (group 3, 39.4%, 95% C.I. 31.8–46.9); Table 1 shows the comparisons before and after focal photocoagulation between groups.

**Table 1:** Variable comparison between groups.

Variable	Group 1 (n = 77)	Group 2 (n = 20)	Group 3 (n = 63)	*p* ^α^	*p* ^β^ (1 vs. 2)	*p* ^β^ (1 vs. 3)	*p* ^β^ (2 vs. 3)
Female gender (%)	51.9	70.0	53.9	0.34	—	—	—
Arterial hypertension (%)	40.3	50.0	49.2	0.5	—	—	—
Proliferative retinopathy (%)	36.3	30.0	19.0	0.08	—	—	—
Baseline visual impairment (%)	51.9	55	28.6	0.01*	1.0	0.005*	0.03*
Age (years, mean ± SD)	57.5 ± 11.7	61.9 ± 8.3	53.8 ± 17.6	0.12	—	—	—
Diabetes duration (years, mean ± SD)	14.5 ± 6.5	13.1 ± 7.4	15.4 ± 6.7	0.75	—	—	—
Fasting glycaemia (mg/dl, mean ± SD)	158.1 ± 62.6	145.2 ± 57.4	184.3 ± 110.7	0.63	—	—	—
Visual acuity before treatment (logMAR, mean ± SD)	0.40 ± 0.27	0.42 ± 0.28	0.27 ± 0.3	0.002*	0.65	0.001*	0.02*
CPT^γ^ before treatment (µm, mean ± SD)	199.8 ± 30.4	166.8 ± 27.9	156.2 ± 20.3	<0.001*	<0.001*	<0.001*	0.16
CFT^¶^ before treatment (µm, mean ± SD)	199.8 ± 30.4	166.8 ± 27.9	156.2 ± 20.3	<0.001*	<0.001*	<0.001*	0.16
Macular volume before treatment (mm^3*^, mean ± SD)	8.08 ± 0.69	7.83 ± 0.52	7.4 ± 0.56	<0.001*	0.12	<0.001*	0.006*
Visual acuity after treatment (logMAR, mean ± SD)	0.40 ± 0.27	0.36 ± 0.29	0.24 ± 0.32	<0.001*	0.43	<0.001*	0.048*
CPT^γ^ after treatment (µm, mean ± SD)	200.4 ± 36.4	168.9 ± 33.7	163.4 ± 26.1	<0.001*	<0.001*	<0.001*	0.70
CFT^δ^ after treatment (µm, mean ± SD)	229.1 ± 28.2	197.5 ± 20.7	194.1 ± 22.8	<0.001*	<0.001*	<0.001*	0.29
Macular volume after treatment (mm^3^, mean ± SD)	7.83 ± 0.56	7.59 ± 0.51	7.27 ± 0.48	<0.001*	0.09	<0.001*	0.006*

α Kruskall-Wallis test.

β Mann-Whitney’s U test.

γ Center point thickness.

δ Center field thickness.

*Statistical difference.

Sixty-nine eyes had baseline visual impairment (43.1%, 95% C.I. 35.43–50.77), which was more frequent in groups 1 and 2 (51/97, 52.6%) than in group 3 (28.6%, p = 0.002, OR 2.77, 95% C.I. 1.41–5.45) and did not differ between groups 1 (51.9%) and 2 (55.0%, p = 0.8). The comparisons of visual acuity and anatomic variables before and after focal photocoagulation within each group (Table 2), showed that that center point thickness and center field thickness increased in group 3, while macular volume decreased in groups 1 and 2.

**Table 2:** Variable comparisons before and after focal photocoagulation, within each group.

Group	Variable	Before	After	p^α^
1	Visual acuity (logMAR, mean ± SD)	0.40 ± 0.27	0.40 ± 0.27	0.842
	Center point thickness (µm, mean ± SD)	199.77 ± 30.44	200.38 ± 36.41	0.480
	Center field thickness (µm, mean ± SD)	233.08 ± 27.51	229.06 ± 28.2	0.471
	Macular volume (mm^3*^, mean ± SD)	8.08 ± 0.69	7.83 ± 0.56	<0.001*
2	Visual acuity (logMAR, mean ± SD)	0.42 ± 0.28	0.36 ± 0.29	0.492
	Center point thickness (µm, mean ± SD)	166.8 ± 27.99	168.9 ± 33.72	0.573
	Center field thickness (µm, mean ± SD)	196.75 ± 16.07	197.5 ± 20.70	0.936
	Macular volume (mm^3*^, mean ± SD)	7.83 ± 0.52	7.59 ± 0.51	0.002*
3	Visual acuity (logMAR, mean ± SD)	0.27 ± 0.30	0.24 ± 0.32	0.256
	Center point thickness (µm, mean ± SD)	156.2 ± 20.31	163.43 ± 26.15	0.006*
	Center field thickness (µm, mean ± SD)	188.46 ± 19.65	194.06 ± 22.86	0.005*
	Macular volume (mm^3^, mean ± SD)	7.40 ± 0.56	7.27 ± 0.48	0.058

^α^ Wilcoxon’s t-test.

*Statistical difference.

**Figure 4:**
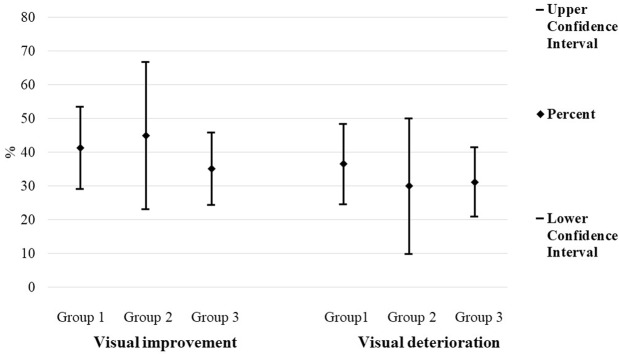
Visual improvement and visual deterioration after focal photocoagulation, in each group.

Mean visual acuity after focal photocoagulation was 20/40 (0.30 ± 0.30 logMAR). Sixty-two eyes had visual improvement (38.8%, 95% C.I. 31.6–46.0): 27 in group1, nine in group 2 and 26 in group 3 (p = 0.6); fifty-three eyes had visual deterioration (33.1%, 95% C.I. 26.15–40.05): 24 in group 1, six in group 2 and 23 in group 3 (p = 0.7, Fig. 4).

Macular volume enlargement, center point thickening, center field thickening and central edema were more frequent in eyes with baseline visual impairment than in eyes without it; the remaining variables had low or inconsistent association strengths. In the logistic regression analysis, the enlargement of the macular volume was the explaining variable of baseline visual impairment when central edema was a predictor (Table 3); however, the effect disappeared when central and paracentral edema were introduced together as predictors.

**Table 3:** Logistic regression in eyes with baseline visual impairment.

Location	Variable	B	S.E.	p	Exp (B)
Central edema	Macular volume enlargement	−0.842	0.38	0.027*	0.43
	Arterial hypertension	−0.569	0.36	0.109	0.57
	Proliferative retinopathy	0.355	0.39	0.363	1.43
	Center point thickening	−0.361	0.59	0.538	0.69
	Female gender	−0.161	0.35	0.647	0.85
	Central edema	−0.375	1.42	0.792	0.69
	Center field thickening	0.124	1.31	0.925	1.13
	Constant	1.255	0.38	0.001*	3.51
Central and paracentral edema	Macular volume enlargement	−0.704	0.38	0.068	0.49
	Arterial hypertension	−0.610	0.36	0.089	0.54
	Central and paracentral edema	0.874	0.54	0.106	2.39
	Proliferative retinopathy	0.368	0.39	0.344	1.44
	Center point thickening	−0.320	0.53	0.548	0.73
	Center field thickening	0.296	0.61	0.631	1.34
	Female gender	−0.114	0.35	0.748	0.89
	Constant	0.558	0.55	0.311	1.75

B = unstandardized coefficient.

S.E. = standard error.

p = p-value.

Exp (B) = Odds ratio of the analyzed variable.

*Statistical difference.

Baseline visual impairment after focal photocoagulation was more frequent in eyes with visual improvement (69.4%) than in eyes without it (30.6%, p =< 0.001, RR 6.26, 95% C.I.3.1–12.6). Male gender showed a trend towards being more frequent in eyes with visual improvement (53.2% vs. 46.8%, p = 0.06), though it was inconsistent. In the logistic regression analysis, baseline visual impairment and male gender were the explaining variables of visual improvement when central edema was the predictive variable; when paracentral and central edema were grouped as a predictive variable the results were similar (Table 4).

**Table 4:** Logistic regression in eyes with visual improvement.

Location	Variable	B	S.E.	p	Exp (B)
Central edema	Baseline visual impairment	−2.442	0.447	0.000*	0.087
	Male gender	0.830	0.408	0.042*	2.293
	Center field thickening	2.064	1.391	0.138	7.878
	Center point thickening	0.869	0.732	0.235	2.384
	Arterial hypertension	0.481	0.414	0.245	1.618
	Central edema	−1.578	1.536	0.304	0.206
	Proliferative retinopathy	−0.185	0.444	0.677	0.831
	Macular volume enlargement	−0.140	0.446	0.754	0.869
	Constant	0.591	0.391	0.131	1.806
Central and paracentral edema	Baseline visual impairment	−2.475	0.455	0.000*	0.084
	Male gender	0.863	0.406	0.034*	2.369
	Arterial hypertension	0.532	0.413	0.198	1.702
	Center field thickness	0.693	0.722	0.337	2.000
	Center point thickness	0.447	0.647	0.489	1.564
	Proliferative retinopathy	−0.259	0.441	0.556	0.772
	Central and paracentral edema	−0.261	0.590	0.658	0.770
	Macular volume enlargement	−0.104	0.449	0.817	0.902
	Constant	0.707	0.613	0.249	2.029

B = unstandardized coefficient.

S.E. = standard error.

p = p-value.

Exp (B) = Odds ratio of the analyzed variable.

*Statistical difference.

## Discussion

Paracentral and central focal diabetic macular edema had similar frequencies of baseline visual impairment, which were higher than in pericentral edema; visual outcome after focal photocoagulation did not differ between groups. Consequently, visual deterioration in eyes with focal paracentral edema (considered center-sparing) was as frequent as in focal center-involving edema.

Baseline visual impairment was less frequent than in a Turkish study (43.1% vs. 60.5%)^10^, probably because over 50% of the subjects in our study had center-sparing edema. The current treatment of center-involving edema with antiangiogenics has replaced photocoagulation: Lang *et al*.^11^ compared ranibizumab combined with photocoagulation against laser alone, in eyes with focal or diffuse macular edema; the proporportion of eyes with visual improvement in the group treated with photocogulation monotherapy (53.5%) was higher than in our study, although their study focused on center-involving edema. In a sensitivity analysis, Wu *et al*.^12^ found that a combined therapy (laser and antiangiogenic) was more effective for improving visual acuity (84.37%) than conventional laser photocoagulation, which produced a worse result than in our study (visual improvement in 0.0%).

Takamura *et al*.^13^ evaluated eyes with focal macular edema measured at the central, inner and outer ring of the optical coherence tomography macular map and found a general improvement in visual acuity by using photocoagulation (n = 27 eyes, p < 0.05, at 12 and 24 weeks from baseline), but did not report the changes in visual acuity according to the location of thickening. The DRCR.net^14^, which evaluated center-involving macular edema, reported visual improvement in 19% and 26% of eyes initially treated with focal photocoagulation using green or yellow laser; that study found that the effect continued up to four months after treatment. None of these studies compared the gain (or loss) of visual acuity according to central, paracentral and pericentral edema location.

All the eyes with paracentral and pericentral focal edema in our study had clinically significant macular edema and risk of moderate visual loss. Vision impairment in paracentral focal edema relates to inflammation and oxidative damage^15^, rather than to the anatomic changes that reduce visual acuity in focal central edema; one reason to evaluate the results at short term, was that focal photocoagulation also induces inflammation, which can impair visual acuity during the first weeks after treatment, an effect that could go unnoticed in the long term.

Even though center field thickness and macular volume have a borderline association with visual acuity, we did not expect to find similar proportions of baseline visual impairment in eyes with central and paracentral edema caused by focal leakage. Browning *et al*. have stated that there are multiple definitions of focal and difusse diabetic macular edema, and that these terms might not explain the variation in visual acuity or outcome after treatment^16^; the definition that we used for focal edema was not included in that paper, despite having being used previously^5^.

By using the angiographic definition of focal edema that we chose, we restricted the selection of cases to those whose edema was caused by leaking mycroaneurysms at the zone of thickening; any patent mycroaneurysm may leak, but only those whose leakege overcome the rate of fluid removal of the retinal pigment epithelium cause retinal thickening. On the other hand, residual thickening that exists after leaking mycroaneurisms close may appear clinically or at optical coherence tomography, without evidence of leaking points at the fluorescein angiography; those zones of thickening require no treatment, provided that they are not the result of a deficient retinal pigment epithelial function, which would have a late fluid accumulation and correspond to the diffuse leakage pattern. A similar definition was used by Perente *et al*.^17^ to treat focal diabetic macular edema with focal photocoagulation.

The difference of the definition of focal edema that we used is that it requires a fluorescein angiography, which has been extensively replaced by optical coherence tomography and optical coherence tomography angiography^18^. The leakage pattern may be irrelevant for practices where antiangiogenics are usually available and affordable, but it is important to make an adequate patient selection in practices where focal photocoagulation is still in use, in order to avoid missing a localized diffuse leakage pattern; those cases could represent a problem because eyes with a diffuse leakage pattern require antiangiogenics, rather than focal photocoagulation.

The Basic and Clinical Science Course of the American Academy of Ophthalmology still reccomends direct laser therapy to leaking mycroaneurysms located between 500 μm and 3000 μm from the center of the macula^8^; in order to be sure that the focal zone of thickening detected clinically or by optical coherence tomography corresponds to a focal leakage pattern, we recommend that those patients have a fluorescein angiography in addition to thicknesss measuring studies.

We chose to treat with focal photocoagulation those eyes with individual leakage arising from microaneurims, because once the leaking sites are closed by laser burns there is no need for monthly retreatments, as in antiangiogenic therapy; we are aware that our results do not apply to eyes with any evidence of edema that is not caused only by micoaneurism leakage, or that have other patterns of optical coherence tomogaphy thickening, such as cystoid, traction or neurosensory detachment.

Closing the microaneurims with focal photocogulation may help to reduce the number of intravitreal injections in patients with other patterns of optical coherence tomography thickening; two studies have dealt with the topic and have found a positive effect^19,20^, but they did not divide their cases according to the location of thickening. We did not add any intravitreal therapy to focal photocoagulation, because our objective was to learn whether focal photocoagulation in center-sparing focal diabetic macular edema had a better result than in cases with center involving focal edema.

The most recent guidelines favor the detection of central thickening, which is easier with optical coherence tomography and makes it unnecessary to diagnose clinically significant macular edema or the fluorescein angiography pattern. Central edema may have thickening outside the central 1,000 µm; an area of edema that starts outside the center and reaches it usually qualifies as a center- involving (regardless of its extension), because the risk of visual loss increases when the foveal photoreceptors separate or degenerate. It is unknown whether baseline visual impairment in eyes with thickening outside the center and at the center existed before this was involved; however, the explaining variable for this dysfunction was macular volume enlargement, rather than the location of thickening.

An increased macular volume shows that there is a wide area of thickening; in eyes with edema that includes both the inner and the outer rings of the macular map, macular volume could increase and cause an extensive retinal dysfunction, which would reduce visual acuity even without affecting the center.

The explaining variable of visual improvement was baseline visual impairment, as the ETDRS reported before optical coherence tomography existed. Its association with an increased volume could involve other changes in diabetic macular edema like alterations of the ellipsoid line^21^ or disorganization of the inner retinal layers. The latter can exist at the macular center and outside it^22^ and has a more consistent association with visual changes in diabetic macular edema than any other optical coherence tomography variable^23^; besides, it reduces the probability of visual improvement after photocoagulation (OR 0.73)^24^.

The strengths of the study are that the patients had no other cause of baseline visual impairment, all the eyes had the risk of visual loss and a focal angiographic leakage pattern, we only used focal photocoagulation and the centering of the macular maps assured adequate measurements of the central 1,000 µm. A potential weakness is the lack of information about macular edema duration, which is difficult to estimate in studies about this topic; another one could be that time domain optical coherence tomography limits the evaluation of the external retinal layers, which could have produced more information. A limitation of the study is the asymmetry of groups, caused by the low prevalence of paracentral edema; this was the result of the frequent coexistence of thickening at the inner ring and at the center, which changed paracentral edema into central.

The patients in group 1 were the last series that received photocoagulation for central edema in our institution, because antiangiogenics offer better anatomic and functional results. Although some clinicians prefer to wait for center involvement to begin treatment, our results show that an equivalent level of visual impairment may develop in eyes with paracentral focal macular edema, and that they need prompt treatment as well.

Rather than returning to the guidelines of the ETDRS, we consider that eyes with paracentral focal diabetic macular edema, measured clinically and with optical coherence tomography, have an elevated risk that needs attention. The definition of center-involvement might need a revision, which preferably includes the central 3,000 μm, especially when there is an augmented macular volume.

In summary, baseline visual impairment was as frequent in paracentral as in central focal diabetic macular edema, and the location of thickening did not change visual results after focal photocoagulation. This treatment for center-sparing focal diabetic macular edema might be reserved for cases with clinically significant thickening caused by mycroaneuysm leakage only, and located more than 1,500 μm away from the foveal center.
